# Six-sigma approach-based visibility graphs

**DOI:** 10.1038/s41598-026-48718-6

**Published:** 2026-04-16

**Authors:** Martin Ferenczi, Alex Kummer, János Abonyi

**Affiliations:** HUN-REN-PE Complex Systems Monitoring Research Group, University of Pannonia, Egyetem u. 10, H-8200 Veszprém, Hungary

**Keywords:** Computational biology and bioinformatics, Mathematics and computing

## Abstract

Visibility graphs transform time series into complex networks where nodes represent time indices and edges encode temporal visibility relationships. While these graphs preserve structural properties of time series, the semantic interpretation of derived network characteristics remains challenging, limiting interpretability and raising questions about what information visibility graphs actually encode. This study enhances visibility graph interpretability using Statistical Process Control (SPC) as an external lens. We construct zone-labeled horizontal visibility graphs (HVGs) that preserve ordinal visibility properties while labeling nodes according to SPC zone classifications (A, B, C zones and control limit violations). Following Six Sigma practices, we quantize the vertical axis into control chart zones and establish explicit correspondences between SPC patterns and graph signatures, including degree distributions, edge weights, motifs, and local communities. The zone-labeled HVG framework successfully maps SPC-charted time series to interpretable network representations. Long runs and trends correspond to skewed in/out degrees and elongated paths, alternation patterns show elevated local clustering, while limit excursions manifest as hubs or articulation points partitioning the graph. This integration provides structured subgraph-level explanations for process alarms, demonstrating that visibility graphs inherently carry actionable information aligned with classical SPC rules, enabling explainable anomaly detection in industrial processes and quality control applications.

## Introduction

Visibility graphs have been extensively applied in time series analysis in various fields, offering a powerful framework for mapping univariate sequences to complex networks whose nodes represent time indices and whose edges encode temporal visibility relationships^1–3^. Network-based time series analysis has achieved considerable progress in recent years, enabling investigations of microscopic and macroscopic behaviors by transforming mono/multivariate time series into network representations^4,5^. The fundamental appeal of the visibility algorithm lies in its simplicity and intuitive geometric interpretation: visibility graphs offer such a representation by recasting a univariate series as a network whose nodes are time indices and whose edges encode an order-based “line of sight”^1^.

For accessibility, we briefly note that visibility graphs (VG/HVG) encode time series as networks by connecting time indices that satisfy a geometric visibility criterion, thereby transforming relative magnitudes and temporal ordering into graph topology^1,6^. Statistical Process Control (SPC), in contrast, provides a widely adopted industrial framework for distinguishing natural variability from special-cause deviations by expressing observations relative to an in-control baseline and discretizing them into $\sigma$-based zones underlying standard alarm rules such as the Western Electric or Nelson criteria^7,8^. Readers seeking broader introductory context on VG/HVG constructions and SPC methodology are referred to foundational and review sources^2,9^.

One of the primary advantages of visibility graphs is their provision of structurally meaningful but often not immediately interpretable representations of time series dynamics^1,6^. The algorithm demonstrates remarkable structural preservation properties, where periodic series are assigned to regular networks, random series to exponential random graphs, and several fractal-like processes have been shown to produce scale-free networks^1,6^. For example, HVGs of random series have exponential degree distributions^10,11^. Beyond global summaries, small patterns in visibility graphs carry discriminative information about the underlying dynamics, providing compact and noise-resistant fingerprints^12^. This natural correspondence between temporal patterns and network topologies has facilitated the development of a natural bridge connecting complex network theory with time series analysis^3,13^.

The framework has been extended in several directions, for example, limited penetrable HVGs for tunable connectivity^14^ and “colored”/degree-vector visibility graphs, where degree-vector VG encodes local rise/fall patterns via node-level degree vectors^15^. Graph-based approaches are now common in the detection of time series anomalies in a more general way^16^. However, the associated network metrics, such as degree distribution, clustering coefficient, or average path length, are considerably less interpretable. Although visibility graphs provide structurally meaningful representations, the semantic interpretation of derived network characteristics often remains problematic^2,4^. Although standard VG/HVG constructions often yield a single static network for an entire series, limiting direct access to evolutionary behavior, several temporal extensions exist (sliding-window/evolving VG, sequential HVG motifs, segment-to-state transition networks)^2,6,12^. These yield snapshots or counts, not a single semantically grounded representation. This limitation raises a fundamental question. Why do these techniques work effectively and what hidden information do visibility graphs actually encode?

The core problem specification thus is the enhancement of interpretability and the elucidation of semantic meaning within visibility graph-derived network metrics. Although visibility graphs successfully perform structural mapping, the practical interpretation of network characteristics frequently proves challenging^1,3^. Centrality and path-based measures can highlight critical intervals in evolving analyses (e.g. anomalous windows in tide gauge records)^6^. However, understanding and interpreting the hidden information within visibility graphs remains an open research area^5^.

The fundamental proposition of this study is to examine the interpretability and information content of visibility graphs through the application of Statistical Process Control (SPC) methodologies. Six Sigma methodology is heavily based on control charts in different phases of the DMAIC framework^7,8^. Graphical representation improves data accessibility and interpretability, facilitating data-driven decision-making and pattern recognition^8^. By applying SPC zones (A, B, C zones and control limit violations) as a semantic layer, we construct zone-labeled visibility graphs that preserve ordinal visibility properties while labeling each node according to its SPC zone classification.

We build a zone-labeled HVG: the nodes retain ordinal visibility, while each node is labeled by its control chart zone relative to an in-control baseline. Following Six-Sigma practice, we quantize the vertical axis into control-chart zones and use the resulting label sequence as a compact diagnostic object (a “network of labels”). We then establish and test rule–structure correspondences (for example, long runs/trends to skewed in/out degrees and elongated paths; alternation to elevated local clustering and characteristic motif frequencies; limit excursions/outliers to hubs or articulation points that split the graph into communities), using subgraphs to explain each detection. We define edge weights as the time span of visibility; average weights estimate ARL between labeled states.

The main contributions of this work are as follows.

1. **Construction:** We provide a reproducible mapping of time series charted with SPC to zone-labeled horizontal visibility graphs (HVG), accompanied by a compact representation of zone sequences for diagnostic purposes.
2. **Rule-structure correspondences:** We establish testable relationships between SPC patterns and visibility graph signatures (degrees, edge weight behavior linked to run-length intuition, motifs, local communities), making detections explainable and interpretable.
3. **Evaluation:** We use two complementary evaluators: (i) a supervised decision tree on metrics + zone characteristics, and (ii) an interpretable rule set on network metrics that maps directly to SPC rules specified by experts or LLM suggested, then programmatically validated (e.g., skewed in/out degree with hub-like betweenness indicating a UCL excursion).
4. **Interpretability:** We provide visualizations of subgraph and neighborhood labels that audit alarm activation mechanisms, complementing standard chart-based evidence with network-theoretic explanations.

In the context of the larger literature, visibility graph analysis has attracted significant attention in recent years, with more than 88% related articles published since 2016, spanning applications in medicine, economics, meteorology, and industrial processes^9^. However, existing approaches rarely connect visibility graph signatures with explicit SPC rules and zone semantics, while the SPC literature seldom employs graph representations to provide structured subgraph-level explanations for process alarms. To the best of our knowledge, no prior work integrates SPC zone labels at node level within HVG to map explicit Western Electric/Nelson rules to graph signatures.

Our objective is to strengthen visibility graph analysis through SPC semantics rather than replacing established SPC rule engines. We demonstrate that visibility graphs already carry actionable information and that classical SPC rules leave recoverable signatures within this representation.

## Methodology

**Fig. 1:**
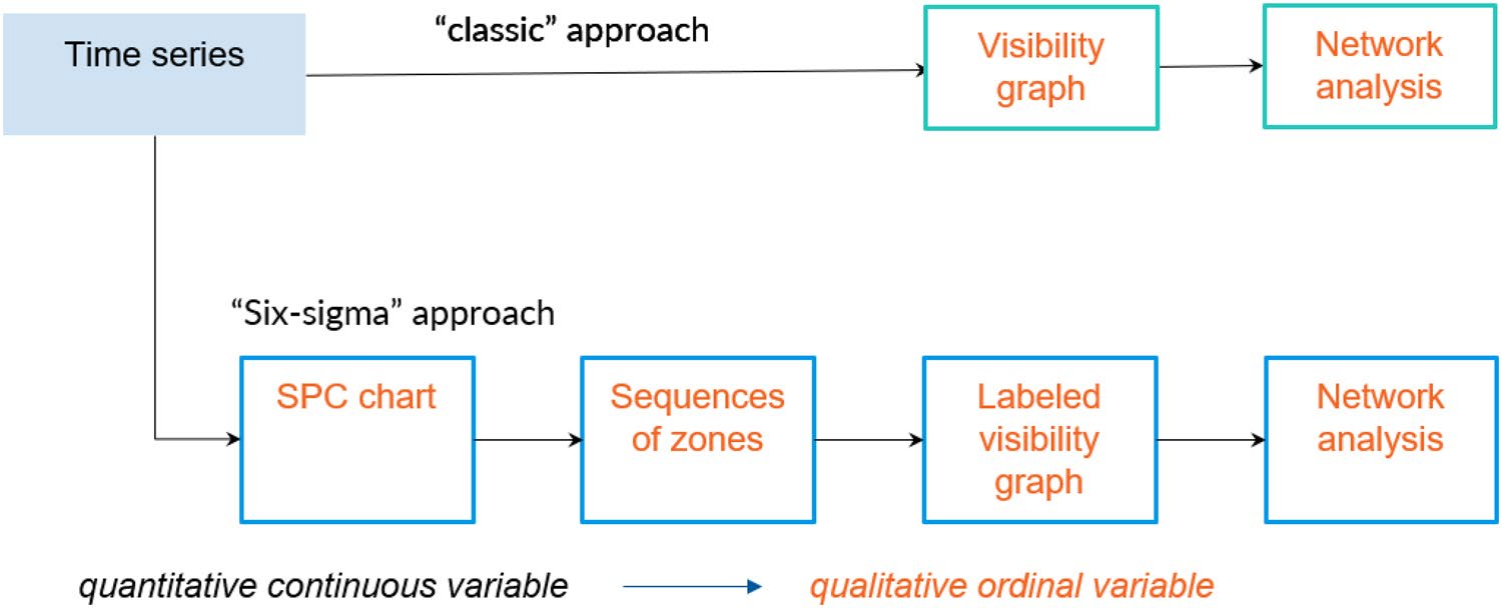
Overall workflow with two parallel branches: classic visibility-graph analysis (top) and Six-Sigma–based, zone-labeled visibility-graph analysis (bottom).

This section formalizes the pipeline shown in Fig. 1, which transforms univariate time series into graph-based structures for structural pattern recognition and SPC-aware anomaly detection. We investigate two branches in parallel: (1) a classic route that applies visibility graph analysis directly to raw data, and (2) a Six-Sigma–based route that first labels each data point with its SPC zone class and then maps the labeled time series to a visibility graph. In both cases, the goal is to perform a leak-free explanation: graph features are computed on the current window, while labels are derived from a strictly future time horizon.

### SPC based visibility graphs

We consider a univariate time series sampled at strictly increasing time instants:

1 $$\begin{aligned} T = \{ (t_i, y_i) \}_{i=1}^{N}, \quad \text {with } t_1< t_2< \dots < t_N. \end{aligned}$$

Each tuple $(t_i, y_i)$ denotes the observed value $y_i$ at time $t_i$. Our goal is to construct a graph-based representation of this series that supports both traditional SPC analysis and structural reasoning via graph properties.

We define the visibility graph of the time series as a graph:

2 $$\begin{aligned} G = (V, E), \end{aligned}$$

where the node set *V* consists of all data points $(t_i, y_i)$, and each node $v_i \in V$ represents a single time–value pair:

3 $$\begin{aligned} v_i \equiv \{t_i, y_i\}. \end{aligned}$$

The edge set *E* is determined by visibility rules that encode structural relations between time points based on their relative heights and positions in the series.

The **horizontal visibility graph** (HVG) provides a method to map time series to complex networks, allowing the application of complex network theory to analyze time series. This simplified variant offers analytical tractability while preserving essential structural information. The HVG algorithm constructs a graph $G = (V, E)$, where each data point $(t_i, y_i)$ corresponds to a node $v_i \in V$. An edge $(v_i, v_j) \in E$ exists between nodes $v_i$ and $v_j$ if:

4 $$\begin{aligned} y_k< \min (y_i, y_j) \quad \forall \, k \text { such that } i< k < j. \end{aligned}$$

**Fig. 2:**
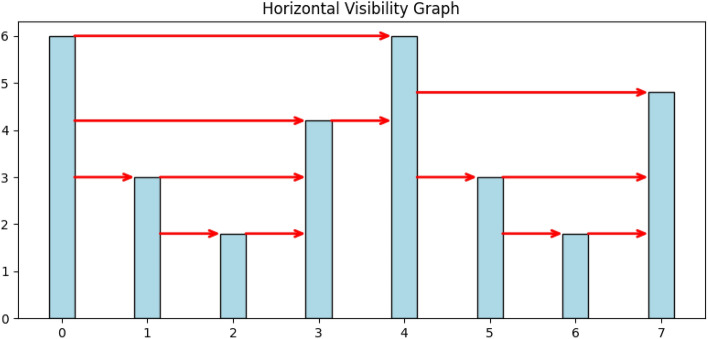
Illustration of a horizontal visibility graph.

This condition implies that the horizontal line segment connecting $v_i$ and $v_j$ must be completely above all intermediate points. This algorithm preserves the temporal ordering of the data points while translating the time series into a network structure that retains essential information about the series’ dynamics (Fig. 2).

A key result regarding HVGs is that the degree distribution of these graphs, when applied to random time series, follows a universal exponential form:

5 $$\begin{aligned} P(k) = \left( \frac{1}{3}\right) \left( \frac{2}{3}\right) ^{k-2}, \end{aligned}$$

which is independent of the probability distribution from which the time series is drawn. This property makes HVGs a powerful tool for distinguishing random time series from nonrandom ones, such as those generated by chaotic processes.

HVGs are also characterized by several notable properties: they are always connected, meaning that each node is linked to at least its immediate neighbors, and they are invariant under affine transformations, ensuring that linear rescaling or translation of the time series does not alter the structure of the HVG. These attributes, coupled with the computational efficiency of the HVG, make it an attractive method for time series analysis.

The HVG method has proven particularly effective in distinguishing between different types of time series dynamics. For example, while random series map into graphs with an exponential degree distribution, chaotic series produce HVGs with distinct topological properties, reflecting the underlying deterministic yet unpredictable behavior of chaotic systems. This capability makes the HVG a valuable tool in various fields, including physics, finance, and beyond, where understanding the dynamics of complex time series is crucial.

Building upon the HVG construction, we now introduce vertex labeling in general terms. We define

6 $$\begin{aligned} G = (V, E, D), \end{aligned}$$

where *V* and *E* follow the HVG rules, and *D* is a finite alphabet of labels attached to the vertices. Labels can encode any discretized attribute of $y_k$. Concretely, in this work we use the SPC-inspired alphabet. This quantization turns the series into a symbolic label sequence; henceforth, we can reason about the process as sequences (and transitions) of zones. The concrete zone definitions are provided below.

7 $$\begin{aligned} D = \{\text {LCL}, \text {A}^-, \text {B}^-, \text {C}^-, \text {C}^+, \text {B}^+, \text {A}^+, \text {UCL}\}. \end{aligned}$$

The labeling function is

8 $$\begin{aligned} \ell : V \rightarrow D, \quad \ell (v_k) = Z(y_k), \end{aligned}$$

where $Z: \mathbb {R} \rightarrow D$ denotes the SPC zoning function that assigns each observation $y_k$ to one of the predefined control-chart zones.

The resulting labeled visibility graph $G = (V, E, D)$ preserves the temporal structure of the original time series through HVG-based edge connections while enriching each vertex with SPC zone semantics through the labeling function $\ell$. This construction serves as a structural carrier that encodes temporal visibility relations through the edge set *E*, while enriching each vertex with SPC semantics derived from process control zones.

This dual representation enables the analysis of both local graph properties (such as degree distribution and clustering) and global SPC patterns (such as runs, trends, and control limit violations) within a unified framework. The construction facilitates leakage-free prediction where graph features are computed in current windows while maintaining strict separation from future labeling horizons, enabling joint use of network topology and symbolic information based on SPC for pattern detection and interpretability.

In our analysis, we extend the classical Western Electric scheme by splitting each of the four rules into direction-aware (+/-) variants relative to the mean, yielding eight rules with unchanged thresholds and run-length counts. This preserves comparability with the original while making upward vs. downward deviations explicit. The rules operate on the standard control-chart zoning around the process mean $\mu$, with boundaries at integer multiples of the standard deviation $\sigma$, which quantifies in-control variability.

**Zone labels used in this paper.** Consistent with the labeled visibility graph, we interpret the previously introduced finite alphabet $D$ as control-chart zone labels with the following semantics:

- $\text {UCL}$: Upper control limit violations $(y_k \ge \mu + 3\sigma )$
- $\text {A}^+$: Zone A above mean $(\mu + 2\sigma \le y_k < \mu + 3\sigma )$
- $\text {B}^+$: Zone B above mean $(\mu + \sigma \le y_k < \mu + 2\sigma )$
- $\text {C}^+$: Zone C above mean $(\mu< y_k < \mu + \sigma )$
- $\text {C}^-$: Zone C below mean $(\mu - \sigma < y_k \le \mu )$
- $\text {B}^-$: Zone B below mean $(\mu - 2\sigma \le y_k < \mu - \sigma )$
- $\text {A}^-$: Zone A below mean $(\mu - 3\sigma \le y_k < \mu - 2\sigma )$
- $\text {LCL}$: Lower control limit violations $(y_k < \mu - 3\sigma )$

These labels match the alphabet $D$ referenced in the labeled graph construction and will be used to analyze sequences and transitions of zones in sliding windows.

The SPC zones and Western Electric (WE) rules are not introduced as an additional anomaly detector, but as an externally validated semantic layer that makes visibility-graph signatures interpretable. In industrial quality control, these rules constitute a widely adopted and auditable vocabulary for “special-cause” deviations: they distinguish isolated extreme excursions (Rule 1) from short clustered extremes (Rule 2), sustained moderate shifts (Rule 3), and long one-sided runs (Rule 4). This categorization is crucial in our setting because different deviation types are expected to leave different topological traces in HVGs (e.g., hub-like spikes vs. distributed connectivity in runs).

Using SPC zoning also provides a principled discretization of the vertical axis based on the in-control baseline $(\mu ,\sigma )$, avoiding ad hoc threshold choices and enabling direct comparability across series, sensors, and operating regimes. Therefore, zones and WE rules serve as the ground-truth semantic reference against which we (i) label windows for supervised evaluation, and (ii) interpret learned or hand-crafted graph features through explicit rule-structure correspondences.

Zone A is defined as the interval where the data points reside within $[\mu + 2\sigma , \mu + 3\sigma )$ above the mean or $(\mu - 3\sigma , \mu - 2\sigma ]$ below the mean. Points in this zone indicate substantial deviations from the process mean, suggesting that the process may be out of control if such occurrences are frequent. Zone B encompasses the regions where the data points fall within $[\mu + \sigma , \mu + 2\sigma )$ above the mean or $(\mu - 2\sigma , \mu - \sigma ]$ below the mean. This zone signifies moderate deviations from the mean and may serve as early indicators of changes in the process. Zone C includes the regions $[\mu , \mu + \sigma )$ above the mean and $(\mu - \sigma , \mu ]$ below the mean. Points within Zone C are considered normal variations that represent minor deviations from the mean.

The most critical zones extend beyond the Upper Control Limit (UCL), defined as $\mu + 3\sigma$, and the Lower Control Limit (LCL), defined as $\mu - 3\sigma$. Points beyond these thresholds (that is, $y_k> \mu + 3\sigma$ or $y_k < \mu - 3\sigma$) signify extreme deviations and are robust indicators that the process is out of control, necessitating immediate investigation.

With the process zones and definitions in place, we can now apply the **Western Electric Rules** to identify and classify deviations in the process. These rules provide a systematic approach to determine when the process might be out of control. In our analysis, we extend the classical Western Electric scheme by splitting each of the four rules into direction-aware (+/-) variants relative to the mean, yielding eight rules with unchanged thresholds and run-length counts:

- **Rule 1 – Points beyond 3**$\sigma$** (direction-aware: UCL/LCL):** One point is more than 3 standard deviations from the centerline, checked separately above (UCL) and below (LCL) the mean.
- **Rule 2 – Two of three consecutive points beyond 2**$\sigma$** on the same side (direction-aware):** Two out of three consecutive points fall in zone A or beyond (i.e., $>2\sigma$ from the mean) on the same side of the centerline.
- **Rule 3 – Four of five consecutive points beyond 1**$\sigma$** on the same side (direction-aware):** Four out of five consecutive points fall in zone B or beyond (i.e., $>1\sigma$ from the mean) on the same side of the centerline.
- **Rule 4 – Eight consecutive points on the same side of the centerline (direction-aware runs):** Eight consecutive points are on the same side of the centerline (zone C or beyond).

Each rule is evaluated separately for values above (+) and below (–) the mean, yielding eight checks in total. This preserves comparability with the original Western Electric scheme, while making upward vs. downward deviations explicit.

The moving window technique is used to analyze time series in a localized and dynamic way. Instead of computing a single visibility graph over the full sequence, the series is partitioned into overlapping windows of fixed size. Each window provides a temporal snapshot over which a labeled visibility graph is independently constructed.

**Fig. 3:**
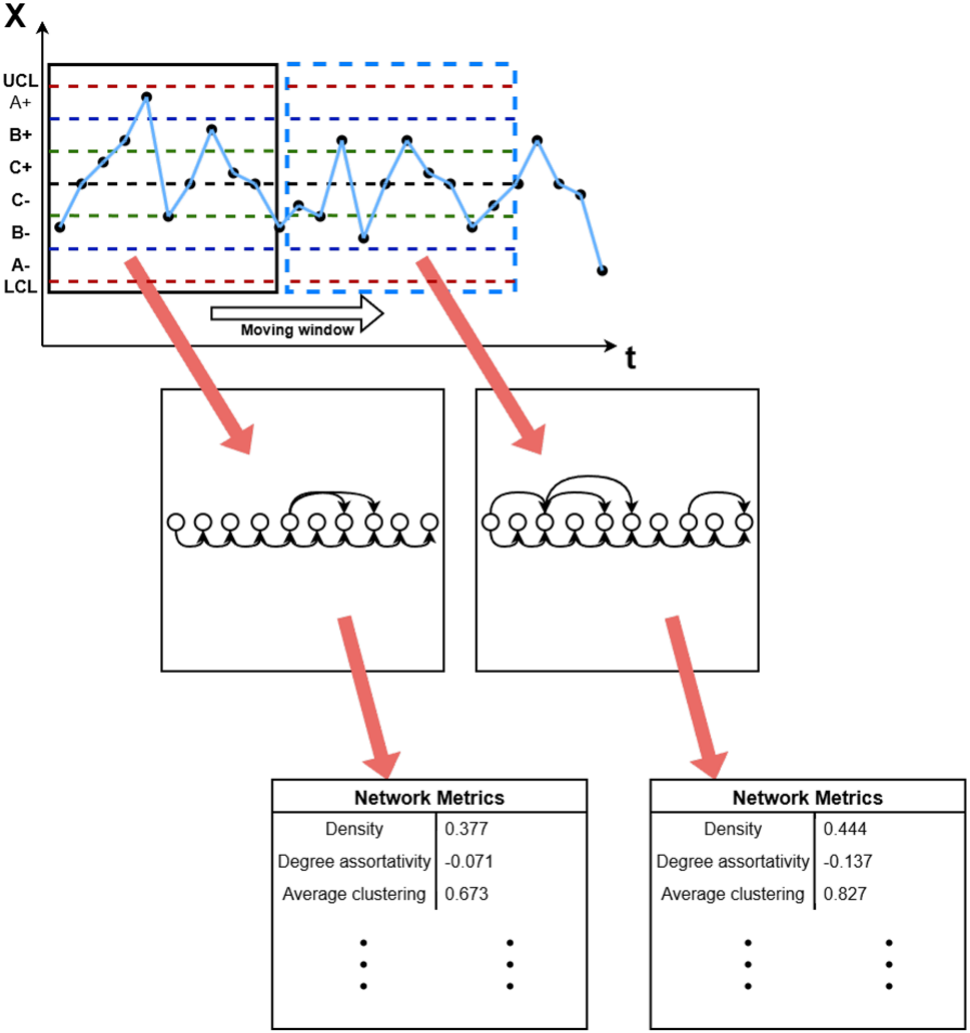
The moving window approach. A fixed-size window slides across the time series. For each window, a labeled visibility graph is generated.

Figure 3 illustrates this procedure. As the window progresses along the series, we generate visibility graphs on each segment and label them with SPC semantics. This approach captures the evolving structure of the data and enables prospective pattern analysis over time.

The sliding-window method is also critical for ensuring leakage-free feature extraction: the graph and all derived features rely solely on the current window, while prediction labels (e.g., anomalies) are tied to a strictly future horizon. As shown in Fig. 3, each window is transformed into an HVG labeled, the basic object for the extraction of features. From this, we compute two complementary families of window-level summaries: (i) visibility-structure metrics (e.g., density, degree assortativity, average clustering), and (ii) SPC-aware label statistics (zone occupancies). These metrics, previewed in the figure callouts, constitute the feature vector and are defined next.

From the labeled visibility graph $G_{vis} = (V, E, D)$ we extract a comprehensive set of network metrics. The feature vector for each window combines structural network properties with zone distribution statistics. We compute several categories of topological features that characterize different aspects of the network structure. Basic connectivity measures include graph density (overall connectivity ratio), average degree (mean node connectivity), and diameter (maximum shortest path length). To assess community structure, we applied modularity optimization to determine the number of communities and measure the modularity score, which quantifies the strength of community separation. Clustering patterns are captured through the average clustering coefficient (local neighborhood connectivity) and transitivity (global clustering coefficient). Finally, we compute degree assortativity, which measures the correlation between the degrees of connected nodes, indicating whether high-degree nodes tend to connect to other high-degree nodes. In addition, we include the empirical distribution of the SPC zone labels within each window, providing direct statistical information on zone occupancy patterns.

### Aggregate state transition networks from visibility graphs

Beyond the structural analysis of individual labeled visibility graphs, we construct an additional aggregate representation that captures the dynamics of SPC zone transitions within sliding windows. This approach transforms the temporal sequence of zone labels into a complementary network structure where nodes represent SPC zones and edges encode transition relationships.

From the labeled visibility graph $G_{vis} = (V, E, D)$, we extract the sequence of zone labels $Z_w = \{z_1, z_2, \ldots , z_m\}$ within each window *w*, where each $z_i \in D$ corresponds to the zone classification of the *i*-th time point.

**Fig. 4:**
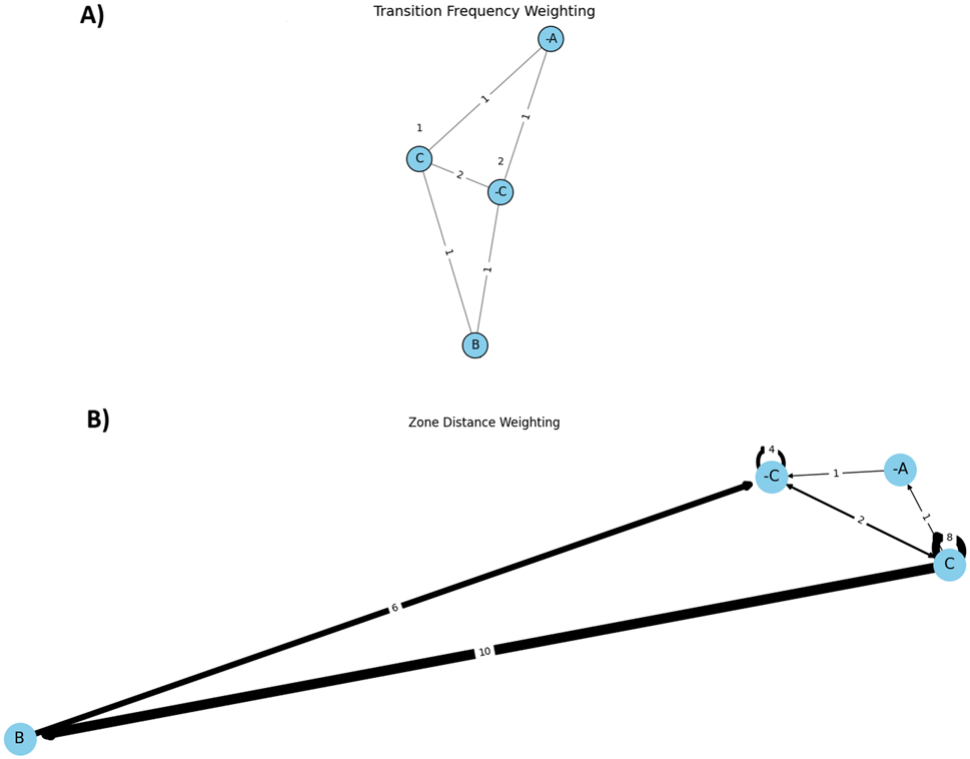
State Transition Network with two complementary weighting schemes. Top: Transition frequency weighting edge thickness and labels indicate raw transition counts; self-loops show within-zone persistence. Bottom: Zone distance weighting edge thickness scales with distance-weighted transitions; dashed edges may mark long jumps.

We construct a **State Transition Network** $G_{state} = (V_{state}, E_{state})$ where $V_{state} = D$ represents the set of SPC zones as nodes, and edges capture transitions between consecutive zones in the temporal sequence. This network operates on two complementary weighting schemes (see Fig. 4):

**Transition Frequency Weighting:** Edges are weighted by the number of direct transitions between zones, highlighting frequently occurring zone changes. Higher edge weights indicate zones that are commonly connected in the temporal sequence. Panel (A) of Fig. 4 shows this weighting scheme, where the edge thickness and the numeric edge labels reflect the raw transition counts; the self-loops indicate the persistence within a zone.

**Zone Distance Weighting:** Edges are weighted by the hierarchical distance between zones in the SPC structure, emphasizing the magnitude of process shifts. Larger weights correspond to transitions that span greater distances in the zone hierarchy (e.g. direct transitions from central zones to control-limit regions). Panel (B) of Fig. 4 illustrates this weighting scheme, where edge thickness scales with distance-weighted transitions and dashed curves highlight long jumps, while edge labels show the corresponding weighted values.

The state transition network provides a compact representation of zone dynamics that complements the structural information captured by the labeled visibility graph. Although the visibility graph preserves temporal ordering and local visibility relationships, the state transition network focuses specifically on the sequence of zone changes and their statistical properties. This dual representation enables comprehensive characterization of process behavior through both structural (visibility-based) and sequential (transition-based) perspectives, forming the foundation for pattern recognition and anomaly detection in the subsequent analysis pipeline.

## Results

### Synthetic data generation and benchmark construction

To systematically evaluate the proposed zone-labeled visibility graph framework and validate the hypothesized correspondences between SPC patterns and graph signatures, we developed a controlled synthetic data generation pipeline that constructs a comprehensive benchmark dataset tailored to this investigation.

Our data generation strategy begins with the creation of baseline time series drawn from a standard normal distribution $\mathcal {N}(0,1)$ with a fixed length of 100 time points. For each series we compute Phase I baseline statistics (mean $\mu$, standard deviation $\sigma$ and the $\pm 1\sigma$, $\pm 2\sigma$, $\pm 3\sigma$ bands including UCL/LCL). These baseline statistics are frozen and not recomputed after injection. The series then serve as the foundation for rule-specific injections that enforce Western Electric (WE) violations in a direction-aware manner (up/down).

The injection methodology operates as follows: for each violation of the target rule, we use frozen $(\mu ,\sigma )$ and modify a randomly chosen window on a randomly chosen side of the mean. To avoid unintended Rule 1 hits, Rules 2–4 clamp injected values strictly below $3\sigma$. Concretely:

- **Rule 1 violations**: a single point is set above $3\sigma$ on the chosen side ($\text {UCL}/\text {LCL}$), that is, $y_i=\mu \pm (3+\delta )\sigma$ with $\delta \in [0.05,0.8]$.
- **Rule 2 violations**: in a 3-point window, two of three consecutive points are placed in Zone A or beyond (i.e., $\ge 2\sigma$) on the same side; the third remains on the same side but below $2\sigma$.
- **Rule 3 violations**: in a 5-point window, four of five consecutive points are placed above $1\sigma$ on the same side (kept $<3\sigma$); the remaining point is on the same side within $[0,1)\sigma$.
- **Rule 4 violations**: eight consecutive points are placed on the same side of the mean using a realistic zone mix on that side (e.g., predominantly C, some B, occasional A), with all values clamped below $3\sigma$.

**Fig. 5:**
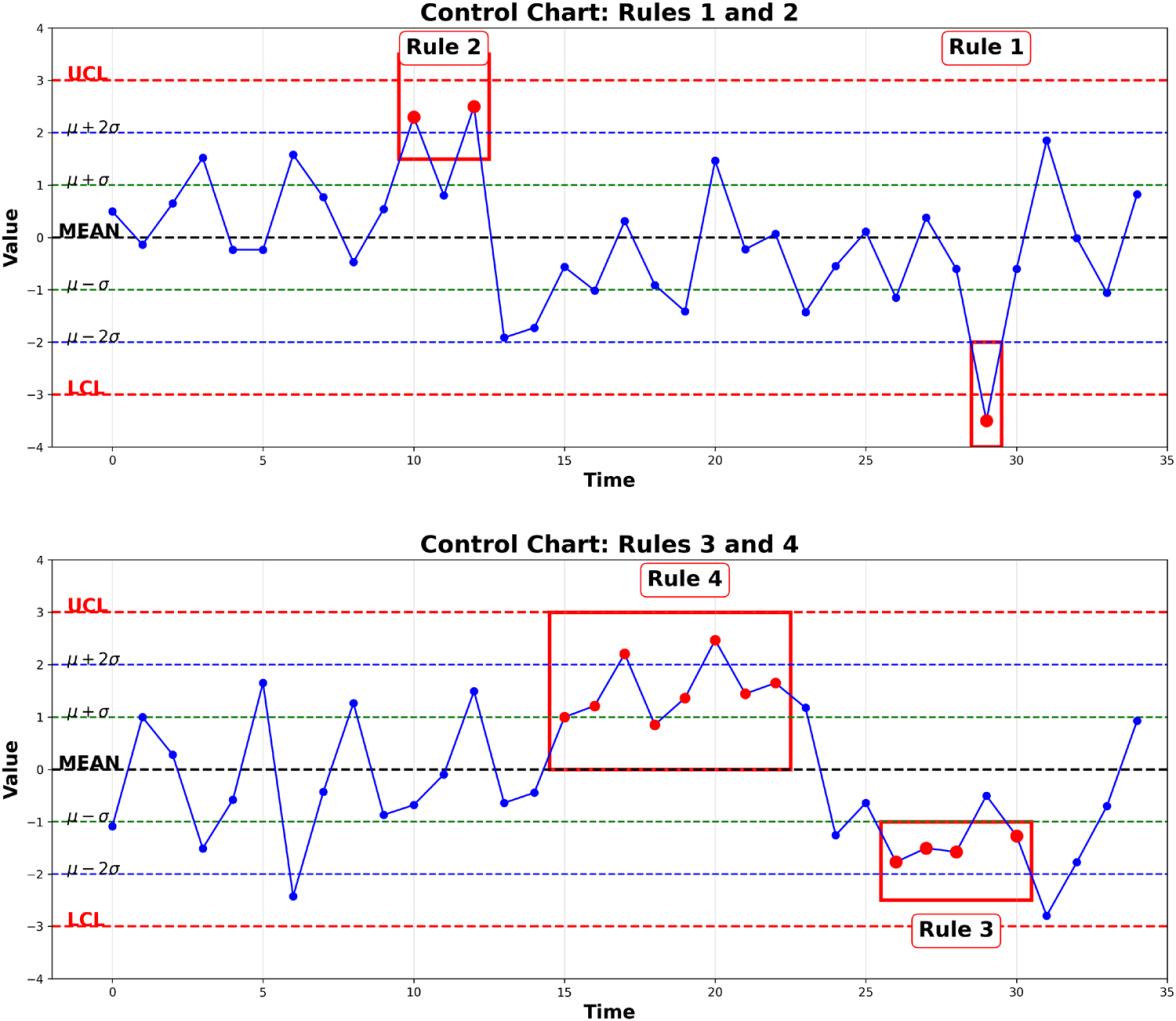
Western Electric rule violations illustrated on control charts. Top: Rules 1 and 2—Rule 1 triggers when a single point exceeds the UCL or falls below the LCL (isolated extreme excursion), while Rule 2 triggers when two out of three consecutive points lie within Zone A on the same side (two-of-three Zone A hits). Bottom: Rules 3 and 4—Rule 3 is triggered when four out of five consecutive points fall within Zone B or beyond on the same side (sustained moderate deviations), while Rule 4 detects eight or more consecutive points on one side of the mean (prolonged single-sided shift).

The examples of control charts in Fig. 5 demonstrate the distinct signatures of the four rules of Western Electric. The top chart shows Rules 1 and 2, where Rule 1 manifests as isolated extreme excursions and Rule 2 appears as two-of-three Zone A hits on the same side. The bottom chart illustrates Rules 3 and 4, showing sustained moderate deviations (four of five above $1\sigma$) and prolonged single-sided shifts (eight in a row).

We do not recompute the statistical parameters after injection; zone boundaries and detection thresholds remain those of the frozen Phase I baseline, ensuring leakage-free labeling and comparability across series.

Through this controlled generation procedure, we construct a balanced benchmark comprising a **control class** (Rule 0, no injection) and **four injected classes** (Rules 1–4). By default, we generate 1000 time series per class, yielding 5000 sequences of length 100. For injected classes, each series contains exactly one embedded violation; the control class contains none. Each sequence is stored with ground-truth labels (rule index and direction), the injection span (start and length), and the frozen baseline statistics, facilitating supervised evaluation of both the classical SPC detection and the proposed visibility graph-based approach.

### Zone-labeled horizontal visibility graph implementation

Having established the synthetic benchmark dataset, we now describe the implementation of our SPC-aware visibility graph framework, focusing on the two core algorithmic components that enable the transformation of time series into labeled network representations.

The SPC implementation uses frozen Phase I control-chart parameters that are computed once per time series and reused for all of its sliding segments. Specifically, for each series we store the sample mean $\mu$ and standard deviation $\sigma$ (with degrees-of-freedom correction), together with the one-, two- and three-sigma bands ($\mu \pm \sigma$, $\mu \pm 2\sigma$, $\mu \pm 3\sigma$; UCL/LCL). These baseline quantities are not recomputed at the segment level.

Given these frozen bands, each value in a segment is mapped to one of eight discrete SPC zones by boundary comparison, following the zone definitions introduced in Section SPC based visibility graphs: LCL (Zone 0), $\hbox {A}^-$ (Zone 1), $\hbox {B}^-$ (Zone 2), $\hbox {C}^-$ (Zone 3), $\hbox {C}^+$ (Zone 4), $\hbox {B}^+$ (Zone 5), $\hbox {A}^+$ (Zone 6), and UCL (Zone 7). This classification yields the alphabet *D* for labeling visibility graph nodes with process control semantics.

Western Electric rule detection is then performed on each segment using these same frozen bands in a direction-aware manner (above/below the mean). Operationally, this is equivalent to testing the corresponding zone memberships: Rule 1 flags any point in Zone 0 or Zone 7 ($>|3\sigma |$); Rule 2 flags windows where two of three consecutive points lie in A or beyond on the same side ($\ge 2\sigma$); Rule 3 flags windows where four of five consecutive points lie beyond $1\sigma$ on the same side (zones B/A or beyond); and Rule 4 flags runs of eight consecutive points on the same side of the centerline (zones C or beyond). The detector returns both the rule index and the direction (up/down).

### Illustrating the information content of visibility graphs through a rule 2 pattern

This case study demonstrates how zone-labeled visibility graphs reveal the structural signature of a short directional spike pattern. We analyze a 10-point window from a 100-observation series where Western Electric Rule 2 (upward) activates: two of three consecutive points fall in Zone $\hbox {A}^+$ on the positive side. Rule 2 is selected as a representative illustrative case because it constitutes a short, localized deviation pattern where both SPC semantics and graph structure interact in a minimal yet expressive way. Unlike Rule 1 (isolated extremes) or Rule 4 (long sustained runs), Rule 2 produces clustered high-zone points within a narrow temporal window, making it particularly suitable for demonstrating how local zone proximity translates into distinct visibility-graph signatures. This allows us to explicitly show the mapping from control-chart semantics to topological features without the confounding effects of prolonged shifts or single-point outliers.

**Fig. 6:**
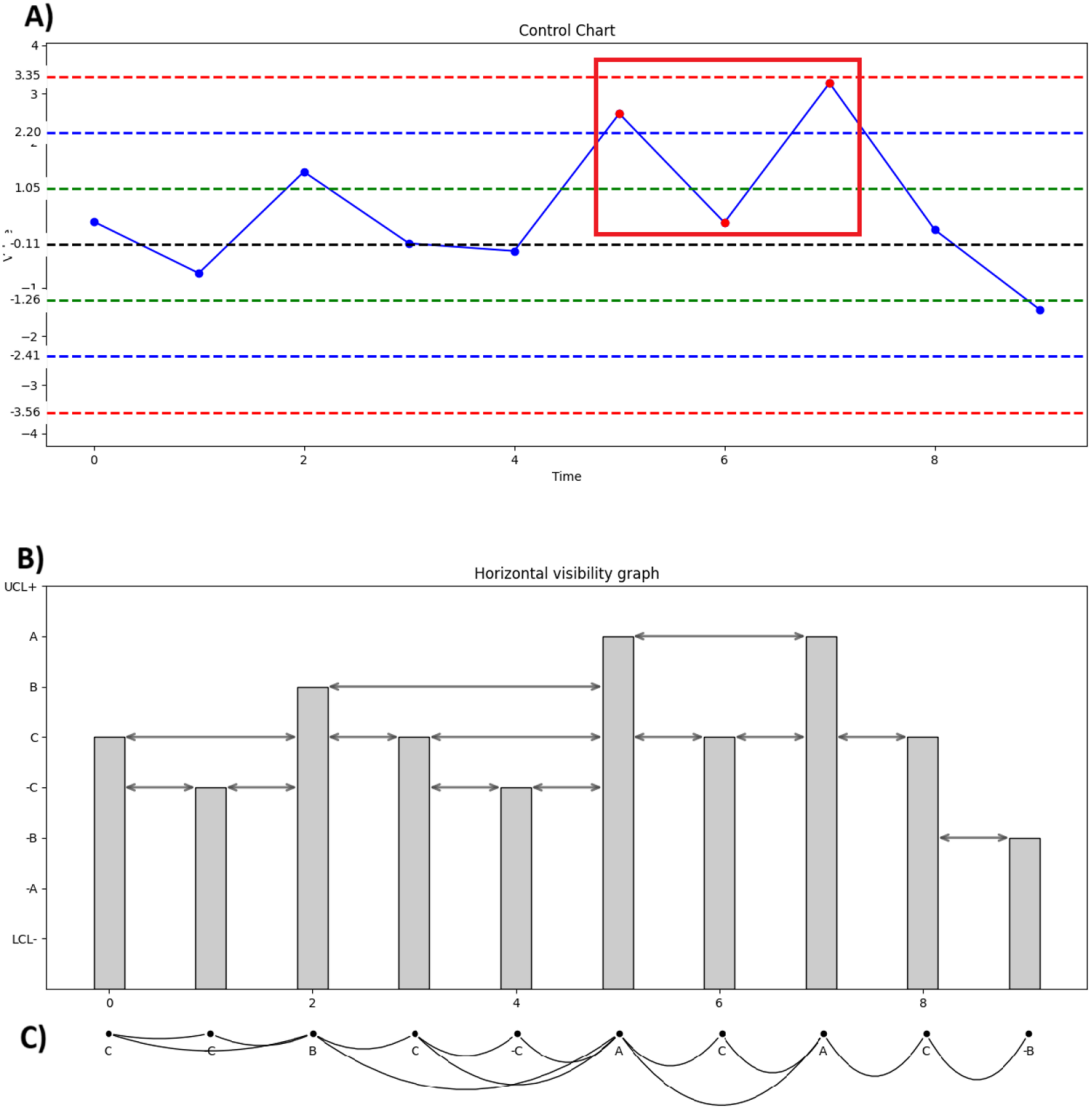
Two-view analysis of the Rule 2 violation. Top: Control chart showing two $\hbox {A}^+$ spikes (red markers) within a 3-point window. Bottom: Combined directed HVG visualization with bar heights, visibility arcs, and neighborhood connectivity view $\hbox {A}^+$ spikes establish long-range visibility arcs (high degree) and dominate local connectivity, while lower zones connect only in short range.

Figure 6 presents complementary perspectives on the Rule 2 violation within the same 10-point window. Panel (A) shows the frozen Phase I bands with the violation highlighted in red. Two points increase to $\hbox {A}^+$ ($\mu + 2\sigma$ to $\mu + 3\sigma$) while the third remains above the mean but below $2\sigma$ a textbook Rule 2 pattern. Critically, this is *not* a sustained shift: the values climb sharply, briefly relax, then spike again.

The question is whether the visibility graph captures this “spike-relax-spike” character. Panel (B) converts height into connectivity: tall spikes “see” far across the window, accumulating edges. The two A nodes act as local hubs of degree 5–7, while the surrounding nodes (predominantly C and B zones on the positive side, with occasional excursions to the negative side) connect only in a short range (degree 2–3). This creates a peaked degree distribution of a few high-degree nodes surrounded by lower-degree neighbors, which is the graph-theoretic fingerprint of a brief excursion.

This signature differs sharply from a Rule 4 (eight consecutive points on one side), where we would expect *uniformly elevated connectivity* to be distributed over many nodes rather than concentrated centers. Panel (C) confirms this: connectivity peaks at the $\hbox {A}^+$ nodes and falls rapidly, while a run would show a sustained high degree throughout the sequence.

**Fig. 7:**
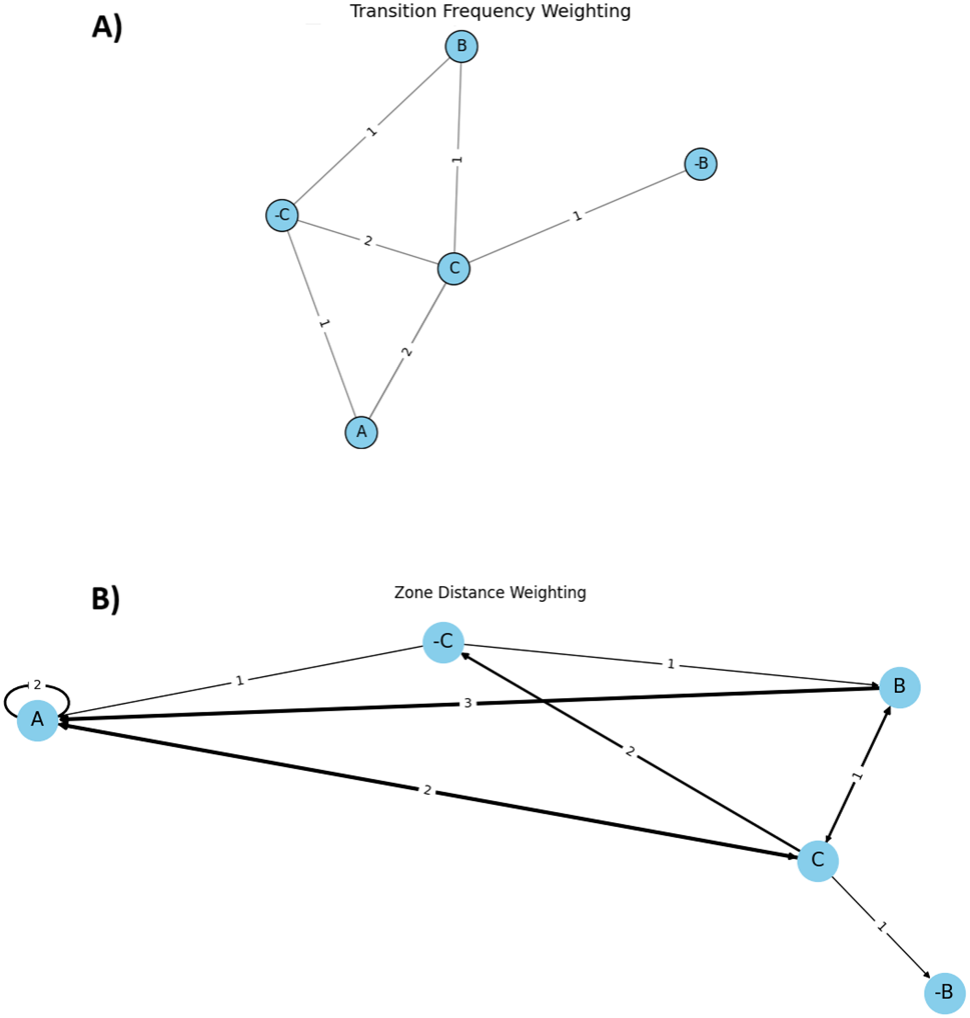
Zone dynamics for the Rule 2 violation. Top: State-transition network (frequency-weighted) shows dominant transitions within the positive side ($\hbox {C}^+$, $\hbox {B}^+$, $\hbox {A}^+$) with brief $\hbox {A}^+$ visits. Bottom: Zone-aggregated directed HVG (edge-count weighted) reveals visibility mass concentration among positive zones and critical $\hbox {A}^+$ self-visibility at distance 2, encoding the “two-of-three” pattern.

The zone dynamics (Fig. 7) provide complementary temporal and aggregate perspectives. Panel (A) shows the temporal sequence of zone changes. Although the window includes points on both sides of the mean, the Rule 2 violation manifests as an escalation on the positive side: $\hbox {C}^+\!\rightarrow$ $\hbox {B}^+\!\rightarrow$ $\hbox {A}^+\!\rightarrow$ $\hbox {C}^+\!\rightarrow$ $\hbox {B}^+\!\rightarrow$ $\hbox {A}^+$. The dominant transition mass concentrates within positive zones, with brief visits to $\hbox {A}^+$ characteristic of the “two-of-three” pattern.

Panel (B) of Fig. 7 reinforces this interpretation: visibility “flows” more heavily between $\hbox {C}^+$/$\hbox {B}^+$ and $\hbox {A}^+$, exactly where the spikes occur. Cross-mean transitions remain sparse, ruling out alternating patterns and confirming the directional nature of the excursion. In particular, the zone-aggregated graph reveals a critical structural signature: $\hbox {A}^+$ exhibits self-visibility with an edge weight of 2, indicating that two distinct $\hbox {A}^+$ nodes in the temporal sequence can “see” each other horizontally. Under normal process conditions, zone A nodes are typically isolated extremes that either do not connect to other A-zone points at all, or are separated by substantial temporal gaps that prevent horizontal visibility. The presence of such short-range $\hbox {A}^+$-to-$\hbox {A}^+$ connections (distance 2) is itself a structural anomaly marker. This low self-distance directly encodes the “two-of-three” Rule 2 pattern: the $\hbox {A}^+$ spikes occur close enough in time that they establish mutual visibility, yet they are separated by the intermediate relaxation point. This graph-theoretic feature therefore provides an interpretable signature distinguishing Rule 2 violations (clustered extremes) from isolated outliers (Rule 1) or sustained shifts (Rule 4), where A-zone self-visibility would either be absent or extend over longer temporal spans.

The combined evidence is unambiguous: the control chart identifies what happened (two out of three $\hbox {A}^+$), the HVG reveals how the pattern manifests structurally (peaked degree at spike nodes), and the zone transition view explains *why* (short excursions into $\hbox {A}^+$, not sustained shifts). All three perspectives converge on the same interpretation using only information available within the 10-point window, without re-computation of baseline statistics. This demonstrates that visibility graphs inherently encode SPC-relevant information in an interpretable form.

### Decision tree classification on network-derived features

Having demonstrated the interpretability of zone-labeled visibility graphs through the focal Rule 2 case study, we now assess the discriminative power of the framework on the full synthetic benchmark. From each labeled visibility graph we compute the metrics defined in Section SPC based visibility graphs (e.g. density, degree assortativity, average clustering, diameter, modularity, and zone distribution statistics), producing a compact feature vector that encodes both structural visibility properties and SPC zone semantics.

Using these features, we train a decision tree to separate the control class (Rule 0) from the eight direction-aware Western Electric violations (Rules 1–4, up/down). To ensure rigorous evaluation, we employ a train-test split: the decision tree is trained on a dedicated training subset and subsequently evaluated on a held-out test set that was not used during model fitting. The tree remains interpretable: splits are thresholds oning transparent correspondences between graph signatures and SPC patterns.

**Fig. 8:**
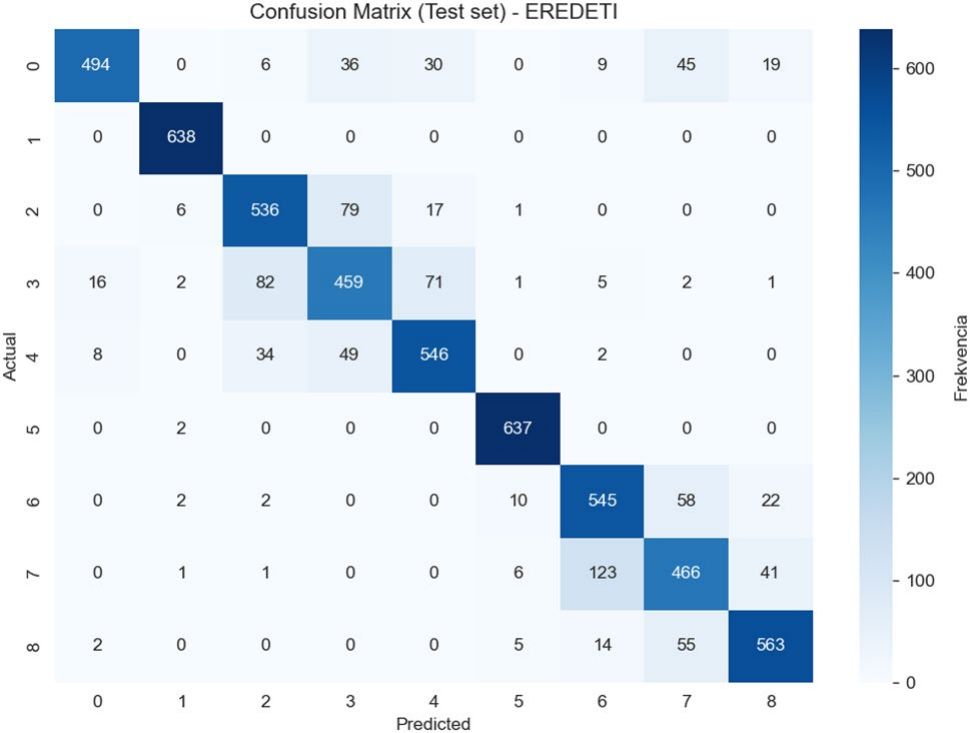
Confusion matrix on the test set (85% accuracy). Classes 0–8 correspond to Rule 0 (control), Rule 1 down/up, Rule 2 down/up, Rule 3 down/up, and Rule 4 down/up. Strong diagonal dominance indicates successful discrimination; systematic off-diagonal entries reflect structural similarities between adjacent rule types.

Figure 8 shows the confusion matrix of the test set, demonstrating performance on previously unseen data, with an overall accuracy of about **85%**. Qualitatively: (i) Rule 1 (UCL/LCL excursions) is easiest to detect, as extreme deviations leave clear graph imprints (e.g. local hubs); (ii) the control class occasionally confuses with directional runs near window boundaries, where sustained but moderate shifts resemble normal variation; (iii) Rule 2 vs. Rule 3 and Rule 3 vs. Rule 4 show systematic cross-confusions consistent with their similar one-sided, short- to long-duration deviations.

In general, the network metrics derived from the zone-labeled visibility graphs carry sufficient discriminative information to recover the semantics of the SPC rules without explicit rule checks, while the structure of the decision tree enables post hoc inspection of the learned thresholds.

To illustrate how the decision tree detects Western Electric violations, we trace the classification path for Rule 2 upward.

For a typical Rule 2 upward violation, the tree follows this path:

1. **Lower than LCL**_**Count **$\le$** 0.5 **$\rightarrow$ FALSE (no LCL violations)
2. **A**_**Count **$\le$** 0.5 **$\rightarrow$ TRUE (limited A-zone points)
3. **Higher than UCL**_**Count **$\le$** 0.5 **$\rightarrow$ FALSE (some UCL vicinity)
4. **B**_**Count **$\le$** 1.5 **$\rightarrow$ FALSE (has B-zone activity)
**-B**_**Count **$\le$** 3.5 **$\rightarrow$ TRUE (limited negative side)
6. **Degree assortativity **$\le$** −0.111 **$\rightarrow$ FALSE (negative assortativity, hub structure)
**-C**_**Count **$\le$** 2.5 **$\rightarrow$ FALSE (limited negative C-zone)
8. **Transitivity **$\le$** 0.638 **$\rightarrow$ FALSE (moderate transitivity)
9. $$\rightarrow$$ **Rule 2 upward**

This path captures the key structural features identified in the case study: negative degree assortativity indicates hub nodes at the A-zone spikes, moderate transitivity reflects localized connectivity rather than sustained runs, and the zone distribution (B_Count, -C_Count) confirms one-sided deviation. The tree learns that Rule 2 differs from Rule 1 (isolated spikes) by intermediate B/C-zone presence, and from Rule 4 (sustained runs) by lower transitivity values.

The decision path demonstrates that graph metrics encode SPC rule semantics in a form learnable by standard classifiers, with interpretable thresholds on network properties replacing explicit pattern-matching logic.

### Discussion

The detailed Rule 2 case study on a 10-point window demonstrates how the same violation manifests consistently across three complementary views: the control chart identifies the two-of-three $\hbox {A}^+$ pattern, the directed HVG reveals peaked degree distributions with high-degree hub nodes at spike locations, and the state transition network shows brief excursions into extreme zones rather than sustained residence. Critically, the zone-aggregated visibility graph exposes $\hbox {A}^+$ self-visibility at distance 2—a structural signature that distinguishes clustered extremes from isolated outliers or prolonged shifts. This multiview convergence demonstrates that the graph structure inherently encodes SPC-relevant patterns in an interpretable form, where each perspective reinforces the diagnostic interpretation available within a single sliding window.

The decision tree classification in 5000 synthetic time series achieves accuracy 85% using features extracted from the zone-labeled visibility graph framework: structural network properties (density, asortativity, modularity, diameter) and zone distribution statistics. This demonstrates that the proposed graph-based representation carries sufficient discriminative power to recover SPC rule semantics without explicit pattern matching algorithms, relying instead on the emergent topological and zonal signatures encoded within the labeled HVG structure. The confusion matrix reveals strong diagonal dominance with systematic confusions between structurally similar rules (Rule 2 vs. Rule 3, Rule 3 vs. Rule 4), consistent with their overlapping one-sided deviation patterns. Although 85% accuracy demonstrates discriminative power, systematic confusions suggest that complementary features such as transition-based metrics from state transition networks or temporal ordering properties may improve separation between adjacent rule types.

While we do not benchmark against classical SPC rule engines, ARIMA-based monitoring, or generic change-point detection methods, our objective in this methodological study is not to outperform established detectors in terms of raw detection accuracy. Instead, we aim to demonstrate that the structural information encoded in HVGs is sufficient to recover canonical SPC semantics without explicitly implementing rule logic. In other words, the contribution lies in the representational layer: showing that graph topology inherently contains the same diagnostic patterns traditionally expressed in control-chart rules.

Classical SPC rule engines directly evaluate predefined thresholds, whereas the proposed framework reveals how these same deviations manifest as topological signatures (e.g., hub-like structures for isolated 3$\sigma$ excursions, distributed high-degree regions for sustained runs). Thus, the value of the approach is interpretability at the structural level rather than replacement of simpler rule-based detection mechanisms.

Several limitations constrain immediate generalization. The benchmark comprises univariate Gaussian series with single embedded violations; real industrial processes exhibit multivariate dynamics, non-stationary trends, autocorrelation structures, and overlapping anomalies that challenge the frozen-baseline assumption. The computational costs of the HVG sliding window construction scale as $O(Nm^2)$, which may limit its applicability to high-frequency real-time monitoring scenarios. Our evaluation relies on balanced synthetic data with known ground truth, whereas industrial datasets typically lack labels and exhibit significant class imbalance, requiring threshold calibration and cost-sensitive learning strategies in deployment.

The integration of SPC semantics with visibility graphs addresses a critical operational gap in regulated manufacturing environments where process deviations require documented root-cause explanations with auditable evidence chains. Traditional control charts provide immediate visual feedback but lack structural reasoning capabilities, while modern graph-based anomaly detectors offer sophisticated pattern recognition, but operate as black boxes. Our framework occupies the middle ground: It leverages the analytical tractability of the HVG construction while preserving the semantic interpretability demanded by Six Sigma practitioners. Zone-aggregated visibility graphs and neighborhood connectivity visualizations enable practitioners to trace alarm mechanisms through subgraph structures, identifying the specific temporal segments and zone transitions responsible for detection a capability unavailable in standard univariate SPC or opaque machine learning pipelines.

## Conclusion

This study enhances the interpretability of visibility graph-based time series analysis by integrating Statistical Process Control zone semantics with horizontal visibility graphs. The HVG construction with SPC zones creates a dual representation that encodes both structural graph properties and explicit process control semantics within a unified mathematical object, where each node preserves ordinal visibility relationships while carrying a zone label derived from frozen Phase I baseline statistics.

Our central contribution demonstrates that visibility graphs are structured carriers of actionable diagnostic information aligned with classical quality control principles. The zone-labeled HVG framework naturally supports leak-free prospective monitoring, as all graph features derive solely from the current sliding window using frozen baseline parameters, ensuring compatibility with Phase II process surveillance protocols.

The framework opens several promising research directions. For anomaly detection, graph-based representation can be extended beyond Western Electric rules to detect novel patterns through unsupervised community detection, motif analysis, or graph kernel methods, enabling discovery of previously unrecognized process signatures. In time series analysis more broadly, the zone-labeling approach generalizes to arbitrary segmentation schemes (quantile-based, entropy-based, or domain-specific thresholds), making visibility graph construction adaptable to diverse monitoring contexts beyond quality control, including financial surveillance, environmental monitoring, and biomedical signal processing.

Future work should address multivariate extensions through multilayer visibility graphs, where each variable forms a layer and cross-layer edges encode dependencies. Handling non-stationarity requires adaptive baseline updating strategies that preserve interpretability while accommodating gradual process drift. Integration with deep learning architectures using visibility graphs as structured input representations for graph neural networks may combine the interpretability of our approach with the representational power of modern ML, particularly valuable for complex fault diagnosis tasks.

By demonstrating that visibility graphs inherently encode SPC-aligned information, this work advances interpretable anomaly detection at the intersection of complex network theory and statistical process control. The zone-labeled HVG framework provides a reproducible methodology for transforming time series into graph representations that preserve both structural visibility properties and diagnostic semantics, enabling transparent and auditable explanations for process alarms. This synthesis positions visibility graphs as interpretable diagnostic tools that bridge the gap between classical SPC and modern data-driven quality assurance, particularly valuable in regulated industries where explainability is essential for operational decision-making.

## Data Availability

The raw data analyzed and the developed code during this study are available on Github: https://github.com/DataCentricSE/Six-sigma-approach-based-visibility-graph.

## References

[1] Lacasa, L., Luque, B., Ballesteros, F., Luque, J. & Nuño, J. C. From time series to complex networks: The visibility graph. *Proc. Natl. Acad. Sci.* **105**, 4972–4975. 10.1073/pnas.0709247105 (2008). 18362361 10.1073/pnas.0709247105 PMC2278201

[2] Stephen, M., Gu, C. & Yang, H. Visibility graph based time series analysis. *PLoS One* **10**, e0143015. 10.1371/journal.pone.0143015 (2015). 26571115 10.1371/journal.pone.0143015 PMC4646626

[3] Gao, Z.-K., Small, M. & Kurths, J. Complex network analysis of time series. *EPL Europhys. Lett.* **116**, 50001. 10.1209/0295-5075/116/50001 (2017).

[4] Zou, Y., Donner, R. V., Marwan, N., Donges, J. F. & Kurths, J. Complex network approaches to nonlinear time series analysis. *Phys. Rep.* **787**, 1–97. 10.1016/j.physrep.2018.10.005 (2019).

[5] Wen, T., Chen, H. & Cheong, K. H. Visibility graph for time series prediction and image classification: A review. *Nonlinear Dyn.* **110**, 2979–2999. 10.1007/s11071-022-08002-4 (2022). 36339319 10.1007/s11071-022-08002-4 PMC9628348

[6] Donner, R. V., Zou, Y., Donges, J. F., Marwan, N. & Kurths, J. Visibility graph analysis of geophysical time series: Potentials and possible pitfalls. *Acta Geophys.* **60**, 589–623. 10.2478/s11600-012-0032-x (2012).

[7] Shewhart, W. A. *Economic control of quality of manufactured product* (D. Van Nostrand Company, 1931).

[8] Montgomery, D. C. *Introduction to statistical quality control* 6th edn. (John Wiley & Sons, 2009).

[9] Najarzadehpour, M., Hosseini, M. & Asgari, S. A review of visibility graph analysis. *IEEE Access* **12**, 73824–73846. 10.1109/ACCESS.2024.3405964 (2024).

[10] Lacasa, L., Luque, B., Ballesteros, F., Luque, J. & Nuno, J. C. From time series to complex networks: The visibility graph. *Proc. Natl. Acad. Sci.* **105**, 4972–4975. 10.1073/pnas.0709247105 (2008). 18362361 10.1073/pnas.0709247105 PMC2278201

[11] Luque, B., Lacasa, L., Ballesteros, F. & Luque, J. Horizontal visibility graphs: Exact results for random time series. *Phys. Rev. E* **80**, 046103. 10.1103/PhysRevE.80.046103 (2009). 10.1103/PhysRevE.80.046103 19905386

[12] Iacovacci, J. & Lacasa, L. Sequential visibility-graph motifs. *Phys. Rev. E* **93**, 042309. 10.1103/PhysRevE.93.042309 (2016). 27176314 10.1103/PhysRevE.93.042309

[13] Silva, V. F., Silva, M. F., Ribeiro, P. & Silva, F. M. Novel features for time series analysis: A complex networks approach. *Data Min. Knowl. Discov.* **36**, 1716–1750. 10.1007/s10618-022-00826-3 (2022).

[14] Wang, M. et al. Exact results of the limited penetrable horizontal visibility graph associated to random time series and its application. *Sci. Rep.* **8**, 5130. 10.1038/s41598-018-23388-1 (2018). 29572452 10.1038/s41598-018-23388-1 PMC5865175

[15] Mori, R., Liu, R. & Chen, Y. Measuring the topological time irreversibility of time series with the degree-vector-based visibility graph method. *Front. Phys.* **9**, 777958. 10.3389/fphy.2021.777958 (2021).

[16] Ho, T. K. K., Karami, A. & Armanfard, N. Graph anomaly detection in time series: A survey. arXiv:2302.00058. (2023). 10.1109/TPAMI.2025.3566620 40315075

